# Efficacy of endophytic bacteria as promising inducers for enhancing the immune responses in tomato plants and managing *Rhizoctonia* root-rot disease

**DOI:** 10.1038/s41598-023-51000-8

**Published:** 2024-01-15

**Authors:** Mona M. Abbas, Walaa H. Ismael, Amira Y. Mahfouz, Ghadir E. Daigham, Mohamed S. Attia

**Affiliations:** 1 Botany and Microbiology Department, Faculty of Science, Al-Azhar University, (Girls Branch), Cairo, Egypt; 2 Soil Microbiology Department, Soil, Water and Environmental Research Institute, Agriculture Research Center, Giza, Egypt; 3 Botany and Microbiology Department, Faculty of Science, Al-Azhar University, Cairo, Egypt

**Keywords:** Microbiology, Plant sciences

## Abstract

Around the world, a variety of crops, including tomatoes, suffer serious economic losses due to the *Rhizoctonia* root-rot disease. Herein, *Bacillus velezensis*, *Bacillus megaterium,* and *Herpaspirillum huttiense* isolated from strawberry *(Fragaria chiloensis var. ananassa)* plants were pragmatic as plant growth promotors for battling the *Rhizoctonia* root rot disease and bringing about defense mechanisms as well as growth promotional strategies in tomato plants*.* These endophytic bacteria demonstrated potent antifungal activity against *R. solani* in vitro along in vivo. Data explained that the isolated endophytic bacteria could produce Indole acetic acid, Gibberellic acid GA, and siderophore as well as solubilize phosphate in the soil. The consortium of (*Bacillus velezensis*, *Bacillus megaterium,* and *Herpaspirillum huttiense*) increased the protection % against *Rhizoctonia* infection by (79.4%), followed by *B. velezensis by* (73.52%), *H. huttiense* by (70.5%), and *B. megaterium* by (67.64%), respectively. There was an increase in soluble proteins and carbohydrates in infected plants treated with a consortium of endophytic bacteria by 30.7% and 100.2% over untreated infected plants, respectively. Applying endophytic bacteria either alone or in combination lowered the level of malondialdehyde MDA and hydrogen peroxide H_2_O_2_ and improved the activities of antioxidant enzymes in both infected and uninfected plants. Also, bacterial endophytes have distinctive reactions regarding the number and concentrations of isozymes in both infected and uninfected plants. It could be recommended the commercial usage of a mixture of targeted bacterial endophyte strains as therapeutic nutrients against *Rhizoctonia* root-rot disease as well as plant growth inducer.

## Introduction

By 2025, there will be around 8 billion individuals on the planet, and by 2050, there will be 9 billion. To provide food for this fast-growing global population, agricultural productivity must rise^1^. Unfortunately, crop loss brought on by pathogen attacks, especially those by fungi^2,3^, is a danger to food security. According to Bramhanwade et al. ^4^, almost one-third of the world's annual crop declines due to plant infections. Phytopathogenic fungi reduce crop productivity by 20–40% per year^5^. Vegetable diseases that result in either total or partial crop loss are a problem for the protection of plants globally^6^. All vegetables have been affected by infections, particularly tomatoes, which yearly cause output losses of between 70 and 95 percent^7^. The tomato (*Lycopersicon esculentum L. Solanum lycopersicon* Mill), which accounts for 14% of global fruit and vegetable production, is the 2nd most significant and valuable Solanaceous vegetable crop after potatoes^8–11^. About 4 million hectares of arable land are cultivated to produce tomatoes worldwide, which produces 100 million tons annually valued at between 5 and 6 billion US$^12^. One of the most significant Solanaceous commodities in Egypt is the tomato, which is grown for both local consumption and exportation^13^. Notably, Egypt ranks among the top 10 tomato-producing countries in the world, producing 6.4 million tons of tomatoes annually on an estimated 181,000 acres^8,14^. Tomatoes are considered a rich source of nutrients, minerals, organic acids, vital amino acids, dietary fibers, and vitamins A and C^15,16^. Because tomato infections severely reduce crop yield, they are seen as being of major economic significance^17,18^. Numerous harmful diseases affect tomato production in both quantity as well as quality^19^. Under the danger posed by global warming and the prevalence of infections, refining crop yield and minimizing the employment of pesticides is an urgent need for the agricultural sector^20^. The tomato crop is susceptible to fungal, viral, nematode, and bacterial illnesses^21^. In Egypt, fungal diseases are among the most hazardous biotic stresses that seriously harm crops^22–24^. Fungal plant pathogens have negative impacts on both the quantity and quality of crops, but these effects can be reversed by nonpathogenic fungi that induce plant biochemical defense^25^. Amongst fungal pathogens, *Rhizoctonia solani* is the most destructive for tomato plants^26^. One of the worst fungi damaging tomato crops is *Rhizoctonia solani* (Khun). This plant pathogenic fungus affects many kinds of hosts and is widely distributed. It causes plant diseases such as collar rot, root rot, damping off, and wire stem^27^. Because spores can live for years, the conventional techniques of controlling disease, such as the application of fungicides in and rotation of crops, have not proven successful. It is critical to create more efficient management practices that ensure the preservation of the environment. In contrast to fungicides, biological control of plant diseases uses antagonist nonpathogenic microorganisms that can reduce the disease's potential dangers on a variety of crops^28^. According to recent studies, the application of natural agents is preferred as a safety method for managing *Rhizoctonia* root rot^29^. The physiological immunity known as "induced resistance" (IR) is a critical defense mechanism against plant fungal infections. IR is elicited by specific environmental stimuli. Pathogenesis-related (PR) proteins and increased phenolic chemicals led to the development of resistance^23^. It is known that endophytic microorganisms can colonize the intracellular areas of higher plant parts without causing visible harm to the plants where they live. They have often been found to be rich in bioactive substances^30^. Endophytes and host plants may cooperate mutualistically to the benefit of both parties' fitness^31^. Endophytic microorganisms may help their host plant survive and give protection by creating a variety of chemicals^32^. Endophytes have the capacity to offer direct chemical defense in plants by producing secondary compounds that hinder pests and dangerous microorganisms^33,34^. Various endophytes were reported to have higher biosynthetic skills because they live and reproduce inside healthy plant tissues and may have undergone gene recombination with the host^35^. Therefore, the main objects of the present work are to (1) Isolate and identify endophytic bacteria from Strawberry plants (2) Evaluate the antifungal activity of these endophytic bacteria against *Rhizoctonia solan*i root rot of tomato in vitro and in vivo, (3) Evaluate photosynthetic pigments, metabolic indicators, protein, and phenolics compounds of tomato, and (4) Recognize the impacts of endophytic bacterial metabolites on oxidative enzymes in tomato plants under pot conditions.

## Materials and methods

### Chemicals and reagents

All chemicals were purchased from Sigma-Aldrich Chemical Co (St. Louis Missouri, 63103, USA). The media were gained from Difco (United Kingdom).

### Isolation and purification of endophytes

The fresh and healthy strawberry plants were collected from Badr City, El-Behera Governorate, Egypt in January 2022. The plant collection and use were in accordance with all the relevant guidelines. All plants were immediately taken to the lab and prepared for future work. The strawberry plants have been washed with tap water to remove surface dust and then rinsed twice with distilled water. Next, the plant materials were immersed in 75% ethanol for 2 min, rinsed with 2% sodium hypochlorite (Na Cl O) for 3 min, and finally washed 3 times with sterile distilled water^36^. The plant parts were checked for any surface contamination, in which 100 µL water from the third rinsing was inoculated on three types of media (Reasoner’s 2A agar, PDA, and nutrient agar). The plates were incubated at 30 °C for 2 days to determine surface sterilization efficacy. The sterilized explants were cut with a sterile blade into 0.5 to 0.3 cm pieces and placed on nutrient agar media. The plates were incubated at 30 °C for 2 days. The isolated endophytic bacteria were imperiled for morphological and microscopic description.

### Quantitative determination of plant growth-promoting substances in liquid culture

Bacterial Endophyte isolates were checked for their quantitative capabilities to produce plant growth-promoting substances. The efficacy of (IAA) production is done using the colorimetric technique^37^. Determination of total Gibberellins production was done using the colorimetric technique^38^. Siderophore production was detected by the CAS plate assay method^39^. The ability of isolates to solubilize phosphate was tested by streaking the isolates in the center of Pikovyskyaya’s agar medium plates containing a known amount of tri-calcium phosphate Ca_3_(PO_4_)_2_. The plates were incubated at 37 ºC for 72 h. Phosphate solubilization was detected by the appearance of a clear halo zone around the streak of endophytic strains^40^. The ability of isolates to solubilize potassium was tested according to Zahra^41^. The formation of hydrogen cyanide (HCN) by the endophytic bacterial isolates was assessed according to the methods of Frey et al. and Daigham et al.^42,43^.

### Source of pathogen

The Pathogen *Rhizoctonia solani* was obtained from the Plant Pathology Institute, Agriculture Research Center, Giza, Egypt. The pathogenic fungus' inoculum was established following the method of Hibar et al.^44^.

### Molecular identification of endophytic bacterial isolates

The endophytic bacterial isolates (L1–L2–S7) were cultured in a nutrient broth medium^45^ and incubated at 28ºC for 48 h. for DNA extraction. Patho-gene-spin DNA/RNA extraction kit afforded by Intron Biotechnology Company; Korea was applied. PCR was operated utilizing two universal primers namely 27F (5′-AGAGTTTGATCCTGGCTCAG-3′) and 1492R (5′-GGTTACCTTGTT ACGACTT-3′). The purified PCR products were reconfirmed using a size nucleotide marker (100 base pairs) by electrophoreses on 1% agarose gel. Purified PCR products were sequenced in the sense and antisense directions using 27F and 1492R primers with the incorporation of dideoxynucleosides (dd NTPs) in the reaction mixture^46^. Sequences were analyzed via the Basic Local Alignment Search Tool (BLAST) from (NCBI) website. Meg Align (DNA Star) software version 5.05 was utilized for the phylogenetic testing of sequences.

### In vitro* antagonistic activity of endophytic bacteria against R. solani by the dual culture assay*

Antagonistic activity test of endophytic bacterial strains (L1–L2–S7) was conducted as illustrated by Rosa et al.^47^with minor modification. PDA media in Petri plates have been streaked individually by a fine line of the isolates (24 h. old) along one end of the plate and incubated at 30 °C for 24 h. Next, along the edge of the plate opposing the bacterial inoculum, a 5-mm-diameter mycelial plug of a 7-day-old culture of *R. solani* was applied. As a negative control, a PDA plate loaded with only a mycelial plug of *R. solani* at one side was employed. All treatments were employed in triplicates and then incubated for 5 days at 30 °C. Lastly, the fungal radial growth was estimated, and inhibition was calculated.

### Antagonistic activity of the endophytic bacterial extract

The bacterial isolates under study (L1–L2–S7) were grown on the PDB broth and incubated on the shaker for 48 h. at 30 °C. After incubation, the samples were centrifuged at 20,000 rpm for 10 min. and the supernatant was filtered through a 0.22 µm microbiological filter. The resulting supernatant was kept in sterilized flasks. A disk of each pathogenic fungus *Rhizoctonia solani* was added to the surface of the extract. Incubation was done at 28 °C for 7 days, and the spread of fungal growth was observed on the surface of the extract.

## Experimental design

Three weeks age tomato seedlings (*Solanum lycopersicum* L. var. 023) were achieved from Agriculture Research Centre, Giza, Egypt. Similar seedlings were planted into plastic pots (40 × 40 cm), encompassing a combination of sand and clay (1: 3 W/W), a total of 6 kg, in a plastic greenhouse. Pots stayed in the greenhouse at Day/night temperature (22/18 °C) and relative humidity (70–85%). After planting, the seedlings were normally irrigated and left for 7 days without treatment. The pots were set up with 6 replicates in a subsequent random order : (T1) Tomato seedlings planted in soil that was recently sterilized (Healthy control); (T2) seedlings planted in *Rhizoctonia solani* -inoculated, soil (Control infected); (T3) Healthy seedlings treated with *B. velezensis*; (T4) Healthy seedlings treated with *B. megaterium*; (T5) Healthy seedlings treated with *H. huttiense*; (T6) Healthy seedlings treated with a combination of (*B. velezensis*, *B. megaterium,* and *H. huttiense* ratio 1:1:1); (T7) Infected seedlings managed with *B. velezensis*; (T8) infected seedlings handled with *B. megaterium*; (T9) infected seedlings treated with *H. huttiense*, and (T10) Infected seedlings managed with a mixture of (*B. velezensis*, *B. megaterium,* and *H. huttiense*). Disease severity was recorded 15 days post-inoculation. After 60 days of inoculation, the biochemical indicators resistance was detected.

### Disease symptoms and disease index

The disease indicators were recorded, and the following equation was used to determine the severity of the disease along with the protection percentage.

Protection % = A–B/A × 100%, where A = PDI in diseased control plants B = PDI in diseased-treated plants as reported by Hashem et al.^48^.

### Biochemical defense indicators

Total soluble carbohydrate content in dry leaves was assessed utilizing the anthrone technique according to Irigoyen,^49^. Also, the content of total protein was measured in the dry leaves^50^. The phenol content of the dry leaves was also estimated^51^. Malondialdehyde (MDA) content in fresh leaf was assessed by the thiobarbituric acid (TBA) method conferring to Hu et al.^52^ with slight modification. Hydrogen peroxide (H_2_O_2_) levels were determined^53^.

### Evaluation of antioxidant enzymes activity

Peroxidase (POD) activity was detected corresponding to Verduyn et al.^54^. The activity of polyphenol oxidase (PPO) and SOD were evaluated affording to methods of Matta and Dimond^55^ and Marklund and Marklund^56^, respectively.

### Isozyme electrophoresis

Peroxidase (POD) and polyphenol oxidase (PPO) isozymes were detected corresponding to Trivedi et al.^57^, while polyphenol oxidase (PPO) isozymes were assessed as stated by Knegt & Bruinsma^58^. Detection of SOD isozymes in fresh leaves was done following the method of Beauchamp & Fridovich^59^.

### Statistical analyses

The obtained results had been imperiled to one-way variance analysis (ANOVA). The significant variances between treatments were demonstrated by CoStat (CoHort, Monterey, CA, USA) utilizing the Least Significant Difference (LSD) test at p < 0.05. The results were provided as means of standard errors (n = 3).

### Plant collection

The plant collection and use were in accordance with all the relevant guidelines.

### Ethical approval

There are no experiments on people or animals in this study.

## Results

**Figure 1:**
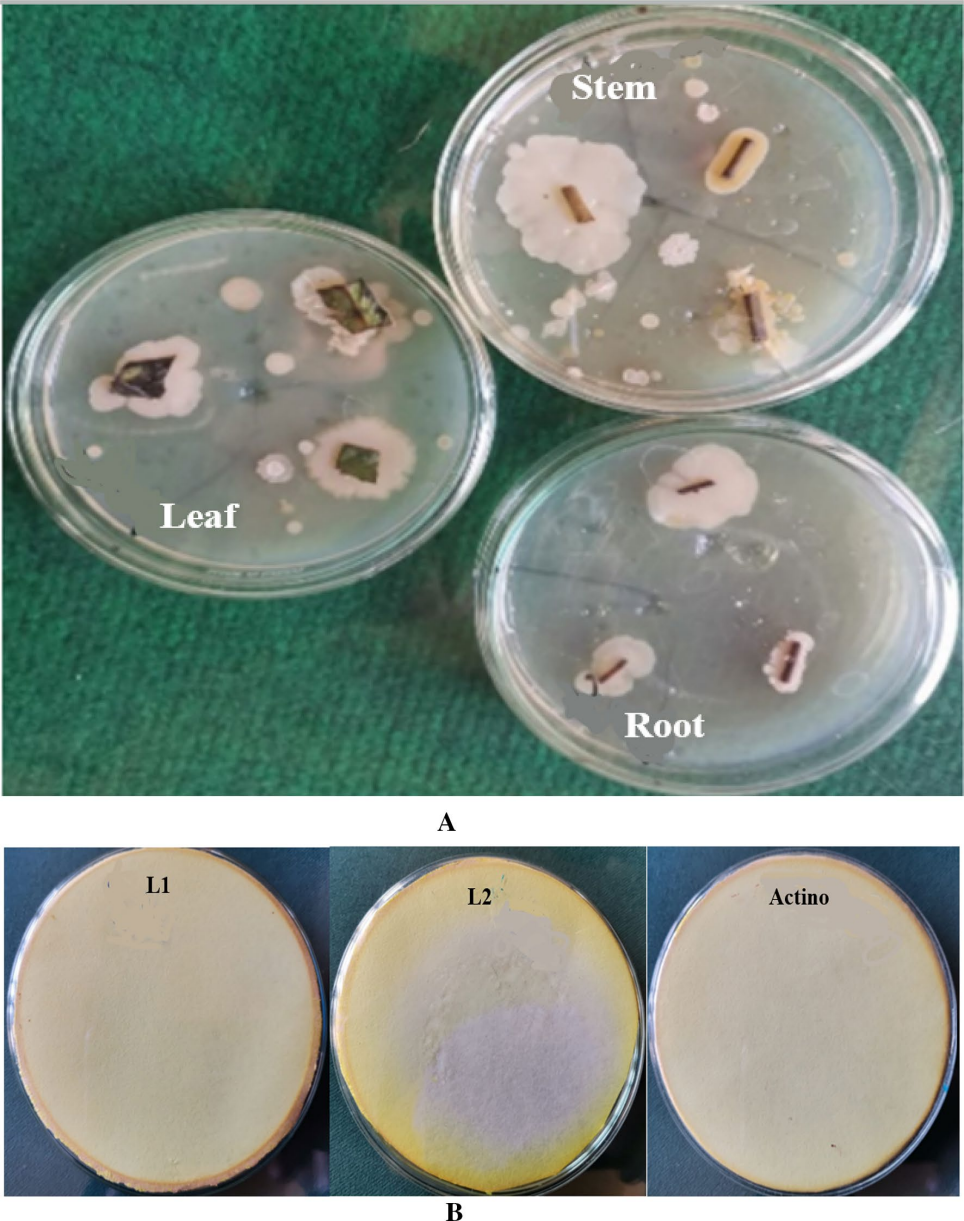
(**A**) Isolation of endophytic bacteria from stem, leaves, and roots of strawberry plants. (**B**) HCN production by endophytic bacterial isolates.

In the present investigation, a total of 31 endophytes were isolated and enumerated from leaves, stems, and roots of Strawberry plants Fig. 1A. All isolates were investigated according to promoting properties. The highest HCN production was recorded by the L1 isolate followed by S7 and then L2 isolate Fig. 1B. The results in Table 1 indicated that none of the isolates were capable of solubilizing potassium. All isolates could produce IAA, where L1 isolate showed a higher production rate (+++), followed by L2 (++) and S7 (++) isolates. The highest potassium solubilization was attained by L2 (+++), followed by S7 (++) and L1 (++) isolates. Moreover, all isolates showed proficiency in siderophores and GA production.

**Table 1:** Determination of plant growth-promoting substances of endophytic bacterial isolates.

Endophytic isolates	K solubilization	IAA	Siderophores	P solubilization	GA	HCN
L1	−	+++	+++	++	+++	+++
L2	−	++	++	+++	++	+
S7	−	++	++	++	++	++

### Molecular identification of bacterial isolates

**Figure 2:**
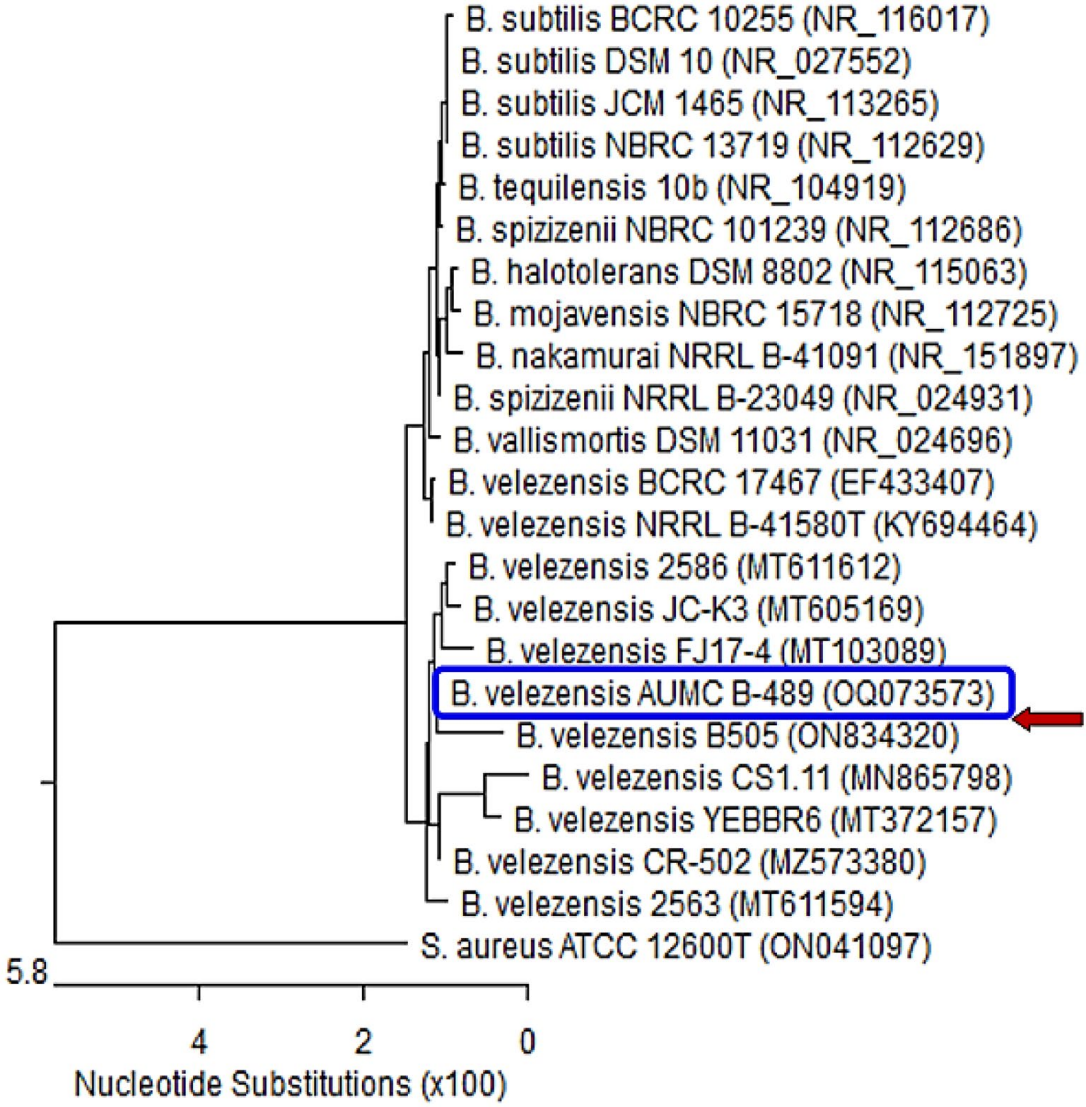
Phylogenetic tree of *Bacillus velezensis* isolate L-1 (arrowed) aligned with sequences of closely related bacterial species. *B. velezensis* L-1 displayed 99.79–100% identity and 99–100% reporting with various strains of the same species with GenBank accession no. OQ073573. B. = *Bacillus* and S = *Staphylococcus*. *S. aureus* represents an outgroup strain.

**Figure 3:**
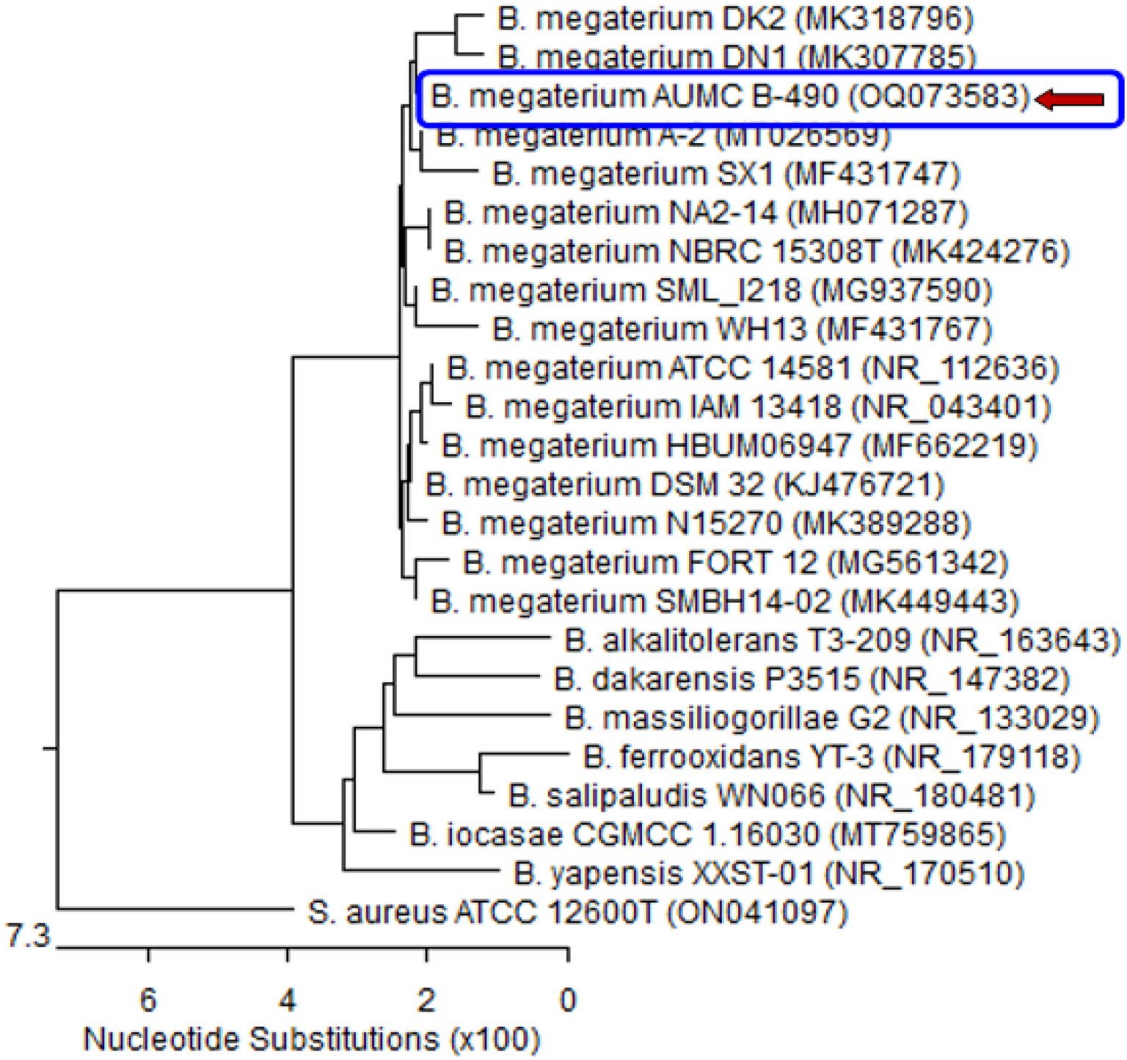
Phylogenetic tree of *Bacillus megaterium* isolate L2 (arrowed) aligned with sequences of closely related bacterial species. *B. megaterium* L2 disclosed 99.72–100% identity and 99–100% reporting with various strains of the same species including the type of material *Bacillus megaterium* ATCC14581 with GenBank accession no. OQ073583. *Staphylococcus aureus* represents an outgroup strain.

**Figure 4:**
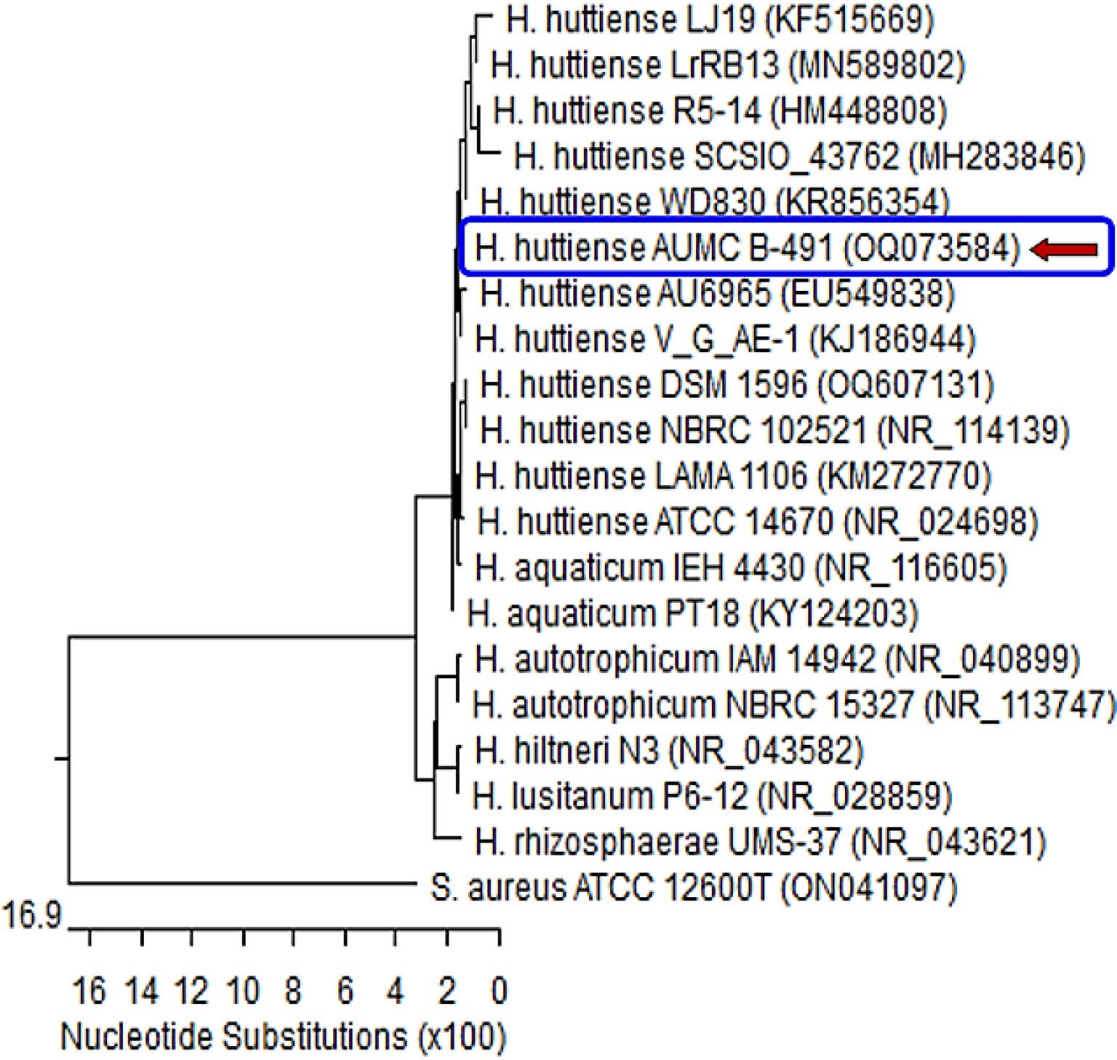
Phylogenetic tree of *Herpaspirillum huttiense* isolate S7 (arrowed) aligned with sequences of closely related bacterial species. *H. huttiense* isolate S7 exhibited 99.50–99.86% identity and 93–100% reporting with various strains of the same species including the type of material *H. huttiense* ATCC14670 with GenBank accession no. NR_024698. *Staphylococcus aureus* represents an outgroup strain.

Based on a mega blast search of the NCBIs GenBank nucleotide database, the closest hit using the sequence of the L1 strain is *Bacillus velezensis.* Figure 2. *B. velezensis* L-1 showed 99.79–100% identity and 99–100% coverage with several strains of the same species with GenBank accession no. OQ073573. On the other hand, the Phylogenetic analysis based on the 16S rRNA gene of *Bacillus megaterium* isolate L2 (arrowed) aligned with sequences of closely related bacterial species. *B. megaterium* L2 showed 99.72–100% identity and 99–100% coverage with several strains of the same species including the type of material *Bacillus megaterium* ATCC14581 with GenBank accession no. OQ073583. *Staphylococcus aureus* represents an outgroup strain Fig. 3. Additionally, based on the 16S rRNA gene of *Herpaspirillum huttiense* isolate S7 (arrowed) aligned with sequences of closely related bacterial species. *H. huttiense* isolate S7 showed 99.50–99.86% identity and 93–100% coverage with several strains of the same species including the type of material *H. huttiense* ATCC14670 with GenBank accession no. OQ073584. *Staphylococcus aureus* represents an outgroup strain, Fig. 4.

### Antagonistic activity of endophytes

**Figure 5:**
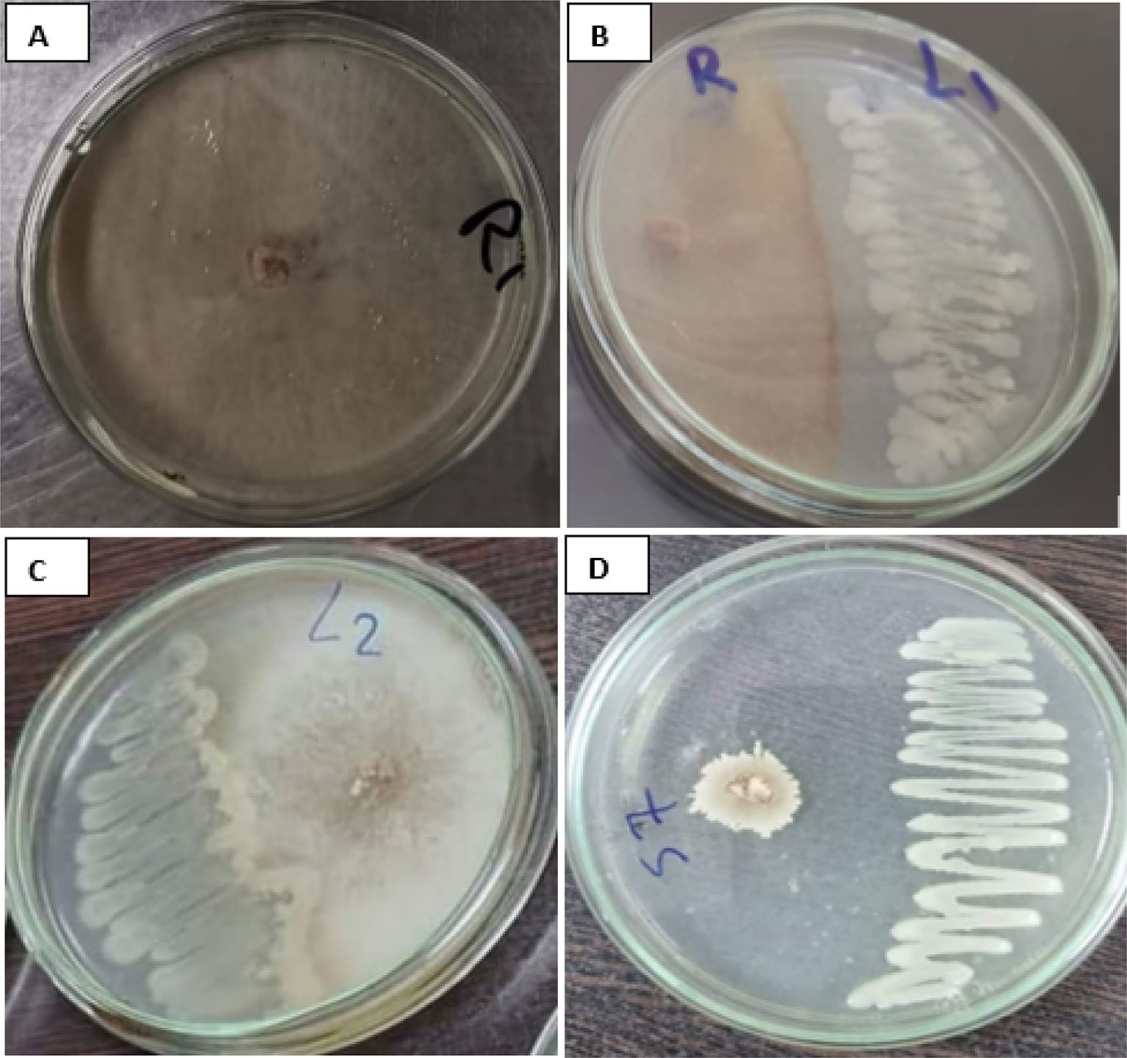
Antagonistic action of endophytic strains versus of *R. solani* using the dual culture method; (**A**) *R. solani* (Control); (**B**) *B. velezensis* (L1) versus *R. solani*; (**C**) *Bacillus megaterium* (L2) versus *R. solani*; (**D**) *Herpaspirillum huttiense* (S7) versus *R. solani*.

**Figure 6:**
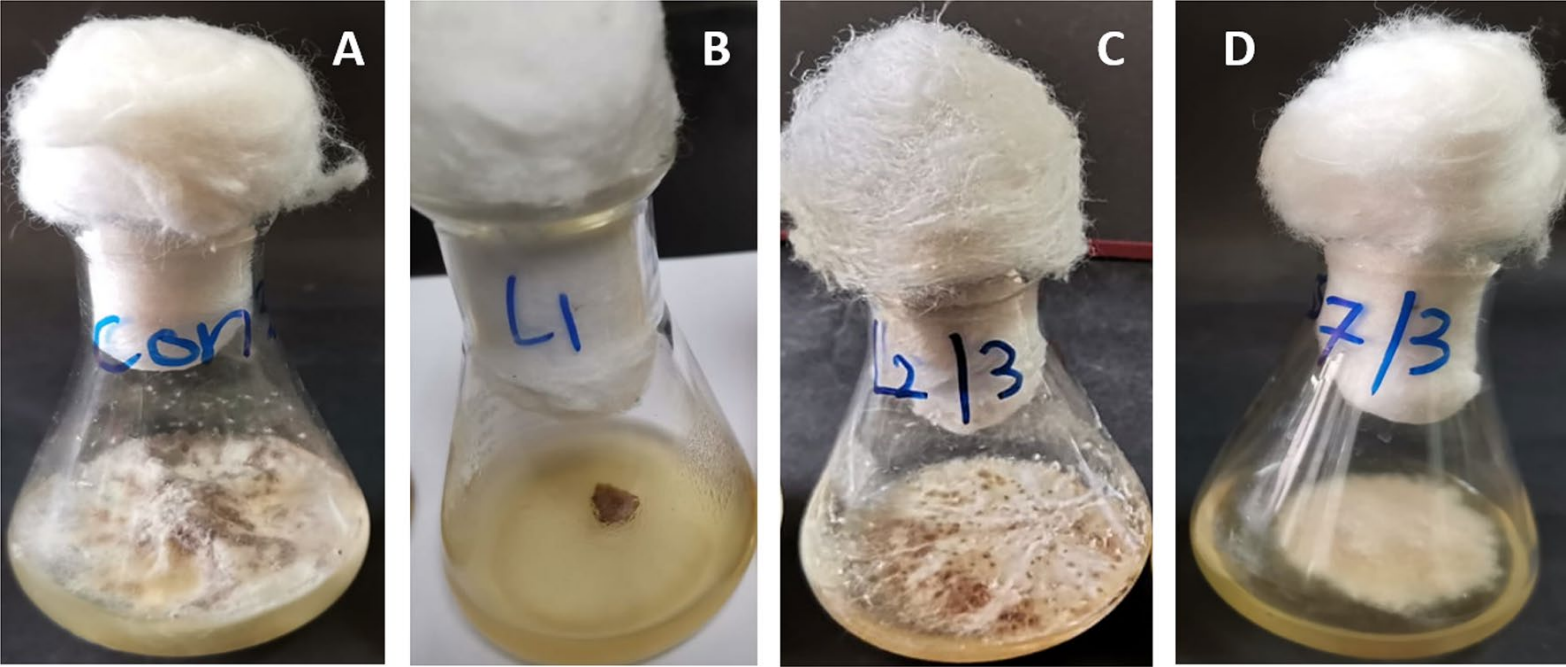
Antagonistic activity of endophytic bacterial extract; (**A**) *R. solani* (Control); (**B**) extract of *B. velezensis* (L1); (**C**) extract of *Bacillus megaterium* (L2); (**D**) extract of *Herpaspirillum huttiense* (S7).

Varying degrees of mycelial growth inhibition of the phytopathogenic fungus *R. solani* using the dual culture assay were observed with antagonistic endophytic bacteria *Bacillus velezensis* L1, *Bacillus megaterium* L2 and *Herpaspirillum huttiense* S7. The results indicated that *Herpaspirillum huttiense* S7 had the maximum inhibitory effect on the mycelial growth of *Rhizoctonia solani* followed by *Bacillus velezensis* isolate L1 and then *Bacillus megaterium* isolate L2. The interactions between antagonistic endophytic bacteria and *R. solani* were shown in Fig. 5. Moreover, the extract of endophytic bacteria under study exerts variable degrees of *R. solani* mycelial growth inhibition, and the most effective was *Bacillus velezensis* isolate L1 followed by *Herpaspirillum huttiense* isolate S7 Table 2 and Fig. 6.

**Table 2:** Percentage of *R. solani* mycelial growth inhibition by endophytic bacterial strains*.*

Endophytic isolates	Mycelial growth inhibition % of* R* *Solani*
*B. velezensis* (L1)	100%
*Bacillus megaterium* (L2)	48
*Herpaspirillum huttiense* (S7)	65%

### Disease symptoms and disease index

**Figure 7:**
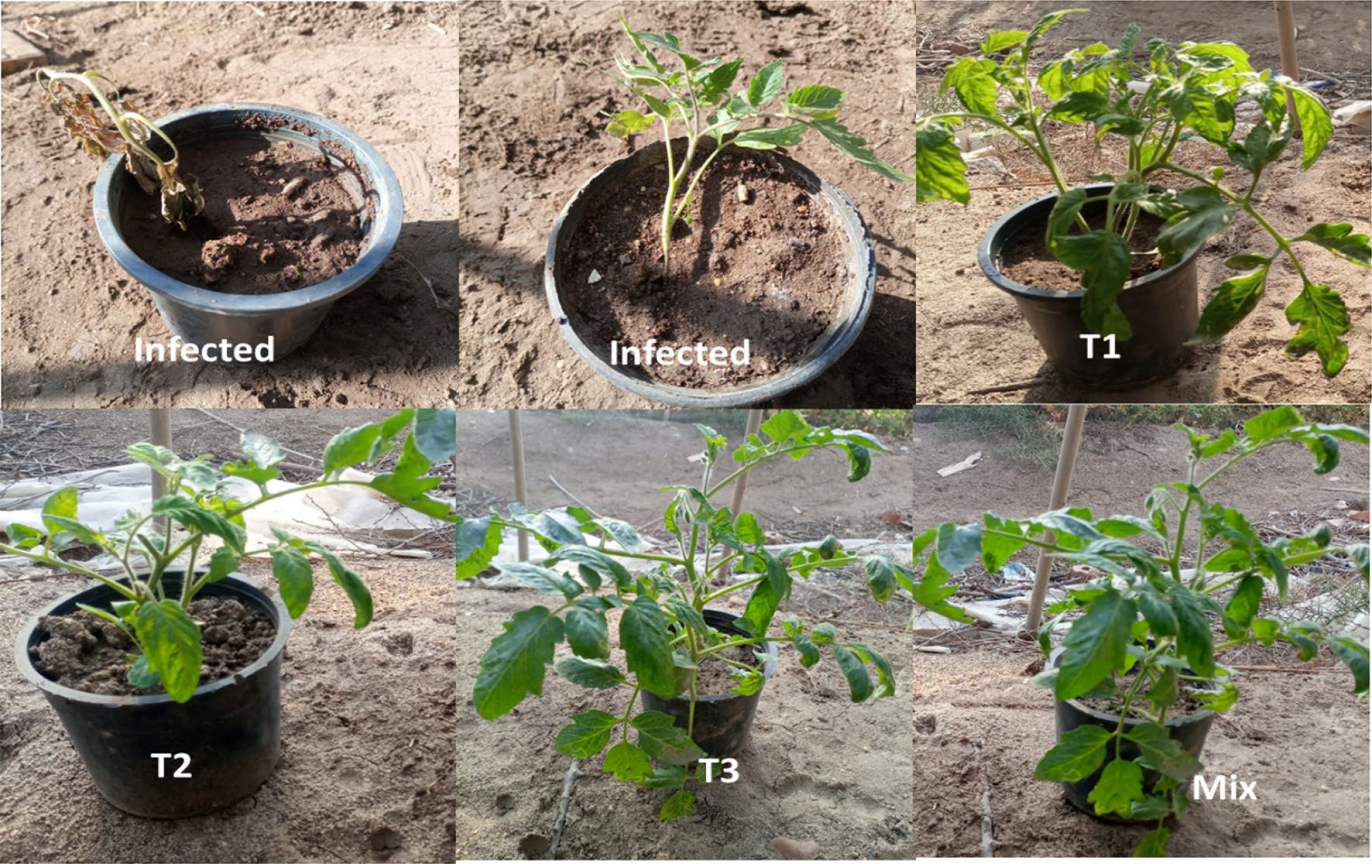
Effect of T1, T2, and T3 on protection of tomato plant against root rot caused by *Rhizoctonia solani* T1 = Infected treated with *B. velezensis*; T2 = Infected treated with *B. megaterium; T3* = Infected treated with *H. huttiense*, Mix = Infected *B. velezensis*, *B. megaterium* and *H. huttiense*).

As shown in Table 3, the disease index reached 85%, due to *R. solani* infection. The results showed that *R. solani* infects the roots of the plant, causing yellowing, root rot, and eventually death. The results in Table 3 and Fig. 7 showed that both *B. velezensis*, *H. huttiense, and B. megaterium* filtrates alone were effective in reducing the severity of the *Rhizoctonia* root rot disease by 22.5%, 25%, and 27.5%, and increase protection by 73.52%, 70.5% and 67.64%, respectively. However, the combining treatment of *B. velezensis*, *B. megaterium,* and *H. huttiense* filtrates was even more effective, with a DI 17.5% and Protection 79.4%.

**Table 3:** Effect of *B. velezensis*, *H. huttiense,* and *B. megaterium* filtrates on root rot disease of tomato plants caused by *Rhizoctonia solani* under pots conditions.

Treatments	Disease symptoms Classes					DI (disease index) (%)	Protection (%)
	0	1	2	3	4		
Control infected	0	0	1	4	5	85	0
Infected treated with *B. velezensis*	4	4	1	1	0	22.5	73.52
Infected treated with *B. megaterium*	4	3	2	0	1	27.5	67.64
Infected treated with *H. huttiense*	6	1	1	1	1	25	70.5
Infected *B. velezensis*, *B. megaterium* and *H. huttiense*)	7	0	2	1	0	17.5	79.4

## Biochemical defense indicators

### Total soluble carbohydrates and protein

**Figure 8:**
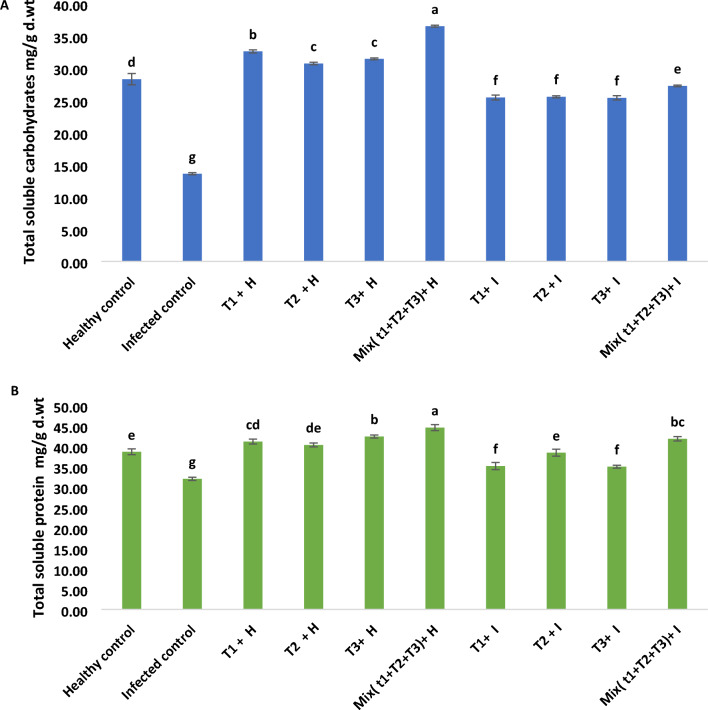
(**A**) Effect of *B. velezensis*, *B. megaterium,* and *H. huttiense* filtrates on of total soluble carbohydrates of healthy and infected tomato plants (**B**) Effect of *B. velezensis*, *B. megaterium,* and *H. huttiense* filtrates on of total soluble protein of healthy and infected tomato plants. T1: Healthy control, T2: Infected control, (T3) Healthy + *B. velezensis*; (T4) Healthy + *B. megaterium*; (T5) Healthy treated with *H. huttiense*,; (T6) Healthy + combination of (*B. velezensis*, *B. megaterium* and *H. huttiense*); (T7) Infected + *B. velezensis*; (T8) Infected + *B. megaterium*; (T9) Infected + *H. huttiense*,; and (T10) Infected + combination of (*B. velezensis*, *B. megaterium,* and *H. huttiense*). The results were represented as (mean ± SD, n = 3), letters authoritative to significant statical assessment.

Results recorded in Fig. 8A declared that infected plants established significant declines in contents of total soluble carbohydrates by 51.9% in comparison with healthy control. Relating the consequence of tested treatments on healthy plants, it was found that all filtrates alone were effective in increasing total soluble carbohydrates by 15.24%, 8.59%, and 11.14%. However, the treatment with mixed filtrates from *B. velezensis*, *B. megaterium,* and *H. huttiense* was even more effective in increasing total soluble carbohydrates by 29.09%. Also, all filtrates alone were effective in increasing total soluble carbohydrates by 86.95%, 87.79%, and 86.65% in infected plants. However, the mixed treatment of all filtrates was even more effective in increasing total soluble carbohydrates by 100.2%. Additionally, results in Fig. 8 B illustrated a substantial decrease in the soluble protein content of *R. solani-infected* plants. Application of all filtrates individual or combination, resulted in, mostly, significant increases in total soluble protein in both healthy and diseased plants. About the effect of  tested treatments on the diseased plants with *R. solani*. It was recovered that mixed treatment with (*B. velezensis*, *B. megaterium,* and *H. huttiense*) filtrates showed a highly substantial rise in the soluble protein contents by 30.07% related to *B. megaterium* 20.8%, *B. velezensis* 9.8% and *H. huttiense* 9.3% when being compared with untreated infected control.

### Phenol content

**Figure 9:**
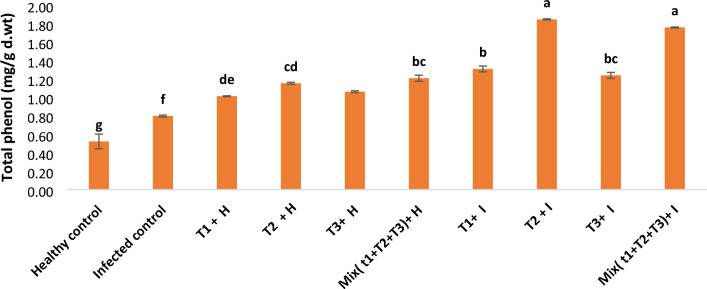
Effect of *B. velezensis*, *B. megaterium,* and *H. huttiense* filtrates on phenol contents of healthy and infected tomato plants. T1: Healthy control, T2: Infected control, (T3) Healthy + *B. velezensis*; (T4) Healthy + *B. megaterium*; (T5) Healthy treated with *H. huttiense*,; (T6) Healthy + combination of (*B. velezensis*, *B. megaterium,* and *H. huttiense*); (T7) Infected + *B. velezensis*; (T8) Infected + *B. megaterium*; (T9) Infected + *H. huttiense*; and (T10) Infected + combination of (*B. velezensis*, *B. megaterium,* and *H. huttiense*). The results were represented as (mean ± SD, n = 3), letters authoritative to significant statical assessment.

As shown from the results in Fig. 9, the phenol content increased by 52.77%, due to *R. solani* infection in comparison with healthy control. Application of all filtrates alone or in combination resulted in, mostly, a significant increase of phenol content in both healthy and infected plants. Regarding the effect of the tested treatment on the affected plants with *R. solani*, the *B. megaterium* treatment showed a significant rise in the content of phenol by 131.2% related to the mixed treatment of *B. velezensis*, *B. megaterium,* and *H. huttiense* by 120.3%, *B. velezensis* by 64% and *H. huttiense* 55.2%, when being compared with untreated infected control.

### ***Oxidative stress (MDA and H***_***2***_***O***_***2***_***)***

**Figure 10:**
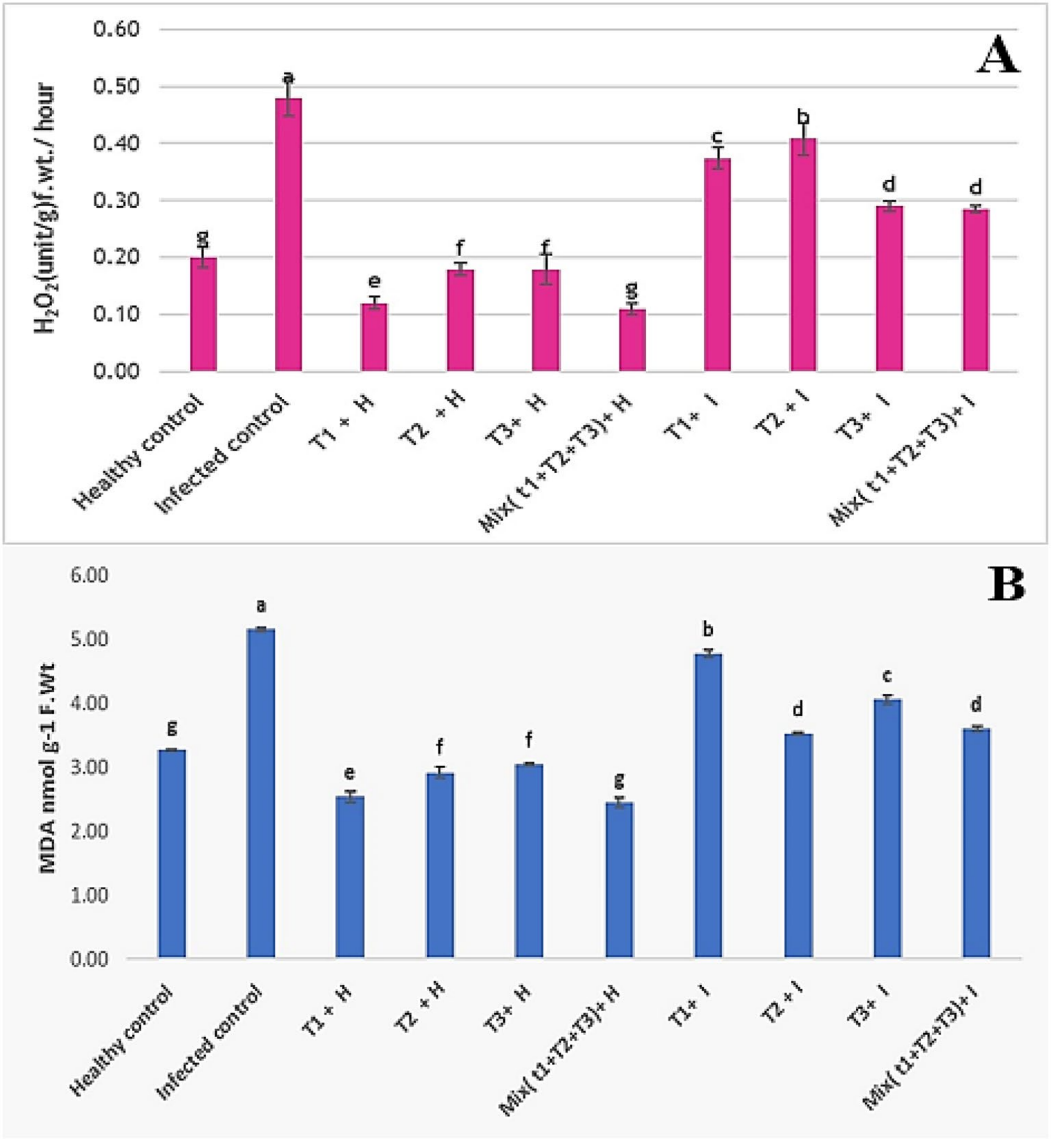
Effect of *B. velezensis*, *B. megaterium,* and *H. huttiense* filtrates on (**A**) MDA and (**B**) H_2_O_2_ of healthy and infected tomato plants. T1: Healthy control, T2: Infected control, (T3) Healthy + *B. velezensis*; (T4) Healthy + *B. megaterium*; (T5) Healthy treated with *H. huttiense*, (T6) Healthy + combination of (*B. velezensis*, *B. megaterium* and *H. huttiense*); (T7) Infected + *B. velezensis*; (T8) Infected + *B. megaterium*; (T9) Infected + *H. huttiense*, and (T10) Infected + combination of (*B. velezensis*, *B. megaterium* and *H. huttiense*). The results were represented as (mean ± SD, n = 3), letters authoritative to significant statical assessment.

As shown in the results represented in Fig. 10A,B, *R. solani* infection accumulated the contents of MDA by 57.6% and H_2_O_2_ by 140%, compared to untreated healthy plants. Application of *B. velezensis*, *B. megaterium,* and *H. huttiense* individually or in combination, resulted in, mostly, a significant decrease in the contents of MDA by and H_2_O_2_. Also, *B. megaterium* exerts a significant decline in the MDA by 37.7% related to mixed treatment of *B. velezensis*, *B. megaterium,* and *H. huttiense* by 30.2%, *H. huttiense* by 21.3% and *B. velezensis* by 7.5%, on infected plants when being compared with untreated infected control. While treatment with a mix of *B. velezensis*, *B. megaterium,* and *H. huttiense* reduces H_2_O_2_ by 40.7% related to *H. huttiense* by 39.2%, *B. velezensis* by 22.1% and *B. megaterium* by 15%, when being compared with untreated infected control.

### Antioxidant enzymes activity

**Figure 11:**
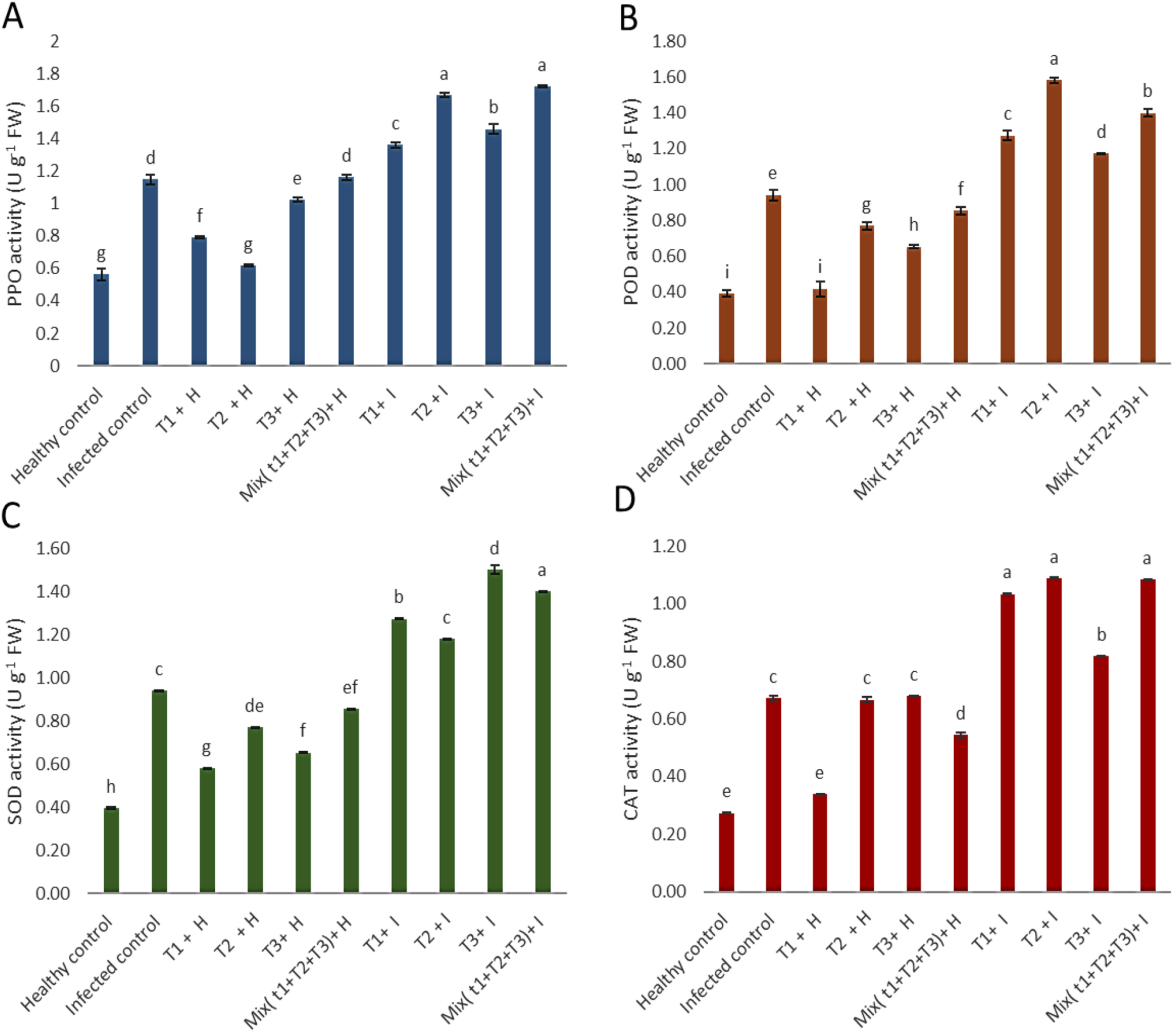
Effect of *B. velezensis*, *B. megaterium,* and *H. huttiense* filtrates on (**A**) PPO, (**B**) POD, (**C**) SOD and (**D**) CAT activities of healthy and infected tomato plants. T1: Healthy control, T2: Infected control, (T3) Healthy + *B. velezensis*; (T4) Healthy + *B. megaterium*; (T5) Healthy treated with *H. huttiense*, (T6) Healthy + combination of (*B. velezensis*, *B. megaterium* and *H. huttiense*); (T7) Infected + *B. velezensis*; (T8) Infected + *B. megaterium*; (T9) Infected + *H. huttiense*; and (T10) Infected + combination of (*B. velezensis*, *B. megaterium* and *H. huttiense*). The results were represented as (mean ± SD, n = 3), letters authoritative to significant statical assessment.

The activities of PPO, POD, SOD, and CAT highly increased in diseased plants linked to healthy control plants Fig. 11. Also, the application of *B. velezensis*, *B. megaterium,* and *H. huttiense* individually or in combination, resulted in, mostly, significant increase activities of antioxidant enzymes PPO, POD, SOD, and CAT, comparing to untreated healthy plants. Concerning the effect of tested treatments, on tomato plants infected with *R. solani*, the results revealed that the mix treatments of *B. velezensis*, *B. megaterium,* and *H. huttiense* filtrates show a highly significant increase in the PPO by 50.1% related to *B. megaterium* 45.5%, *H. huttiense* 27.1% and *B. velezensis* 18.5%. For POD, it was found that *B. megaterium* shows a highly significant increase of 67.81% followed by mixed treatment of *B. velezensis*, *B. megaterium,* and *H. huttiense* by 48.64%, *B. velezensis* by 35.2%, and *H*. *huttiense* 24.8%. Regarding the effect of tested treatments on tomato plants infected with *R. solani*, the results indicated that *H. huttiense* 27.3% shows a highly significant increase in the SOD by 59.6% related to mixing treatment of *B. velezensis*, *B. megaterium,* and *H. huttiense* by 48.94%*, B. velezensis* 35.4%, and *B. megaterium* 25.53%. For CAT, it was found that *B. megaterium* shows a highly significant increase of 62.16% followed by mixed treatment of *B. velezensis*, *B. megaterium,* and *H. huttiense* by 61.43%, *B. velezensis* by 53.92% and *H. huttiense* 21.71%, comparing to untreated infected plants.

### Superoxide dismutase (SOD) isozymes

**Figure 12:**
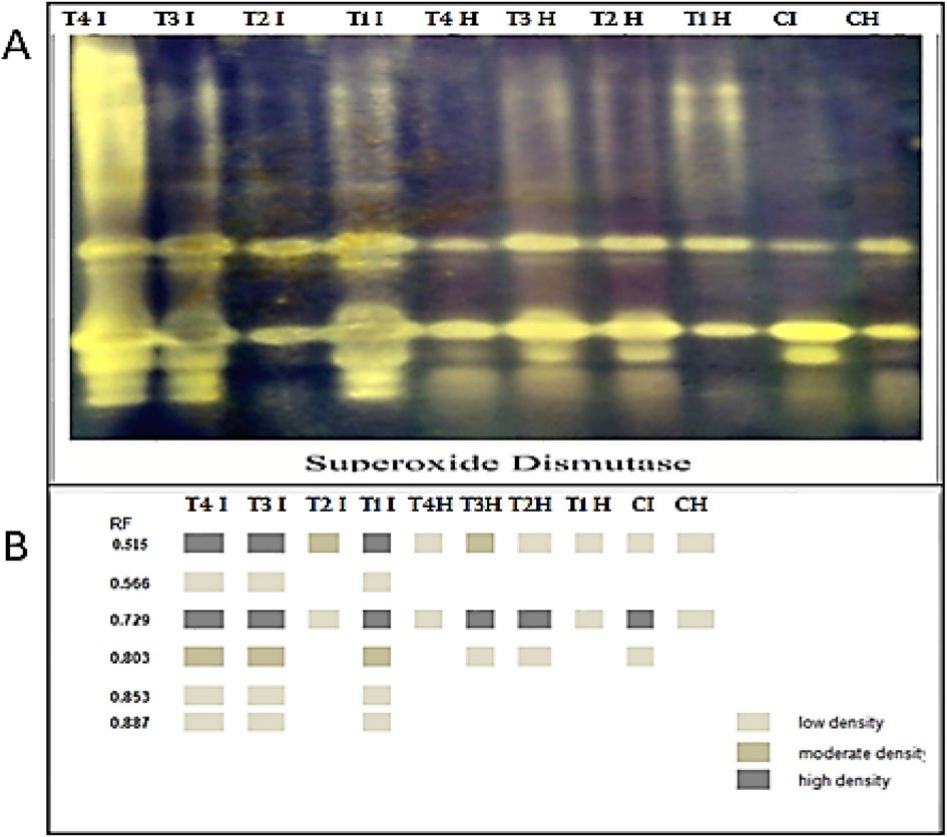
Consequence of *R. solani* infection, application of (*B. velezensis*, *B. megaterium,* and *H. huttiense)* and their impacts on tomato plants on (**A**) SOD isozyme and (**B**) Ideogram analysis of SOD isozyme of tomato plants.

Seven SOD isozymes were noticed on native PAGE in Fig. 12 and Table 4 at Rf 0.515, 0.566, 0.571, 0.729, 0.803, 0.853, and 0.903. Infected tomato plants with *R. solani* exhibited substantially overexpressed SOD, which was detected in three bands, two low and one dense at Rf 0.515 and 0.803 and 0.729 respectively. Under infection circumstances, the application of a mixture from *B. velezensis*, *B. megaterium,* and *H. huttiense*, *H. huttiense,* and B. *velezensis* treatments verified the similar 6 bands at the identical Rf. and came next the *B. megaterium* treatment which confirmed one moderate band at Rf 0.515 and one low band at Rf 0. 729.

**Table 4:** Isomers of SOD enzymes (+/−) and their Retention factor (Rf) in response to *R. solani*, application of (*B. velezensis*, *B. megaterium,* and *H. huttiense)* and their interactions on tomato plants.

RF	T4 I	T3 I	T2 I	T1 I	T4 H	T3 H	T2 H	T1 H	CI	CH
0.515	+++	+++	++	+++	+	++	+	+	+	+
0.566	+	+	−	+	−	−	−	−	−	−
0.571	−	−	−	−	−	−	−	−	−	−
0.729	+++	+++	+	+++	+	+++	+++	+	+++	+
0.803	++	++	−	++	−	+	+	−	+	−
0.853	+	+	−	+	−	−	−	−	−	−
0.903	+	+	−	+	−	−	−	−	−	−

CH = Healthy Control, CI = Infected control, T1H = Healthy + *B. velezensis*; T2H = Healthy + *B. megaterium*; T3H = Healthy + *H. huttiense*, ; T4H = Healthy + combination of (*B. velezensis*, *B. megaterium* and *H. huttiense*), T1I = infected + *B. velezensis*; T2I = Infected + *B. megaterium*; T3I = Infected + *H. huttiense*, ;and T4I = Infected + combination of (*B. velezensis*, *B. megaterium* and *H. huttiense*).

### Peroxidase (POD) isozymes

**Figure 13:**
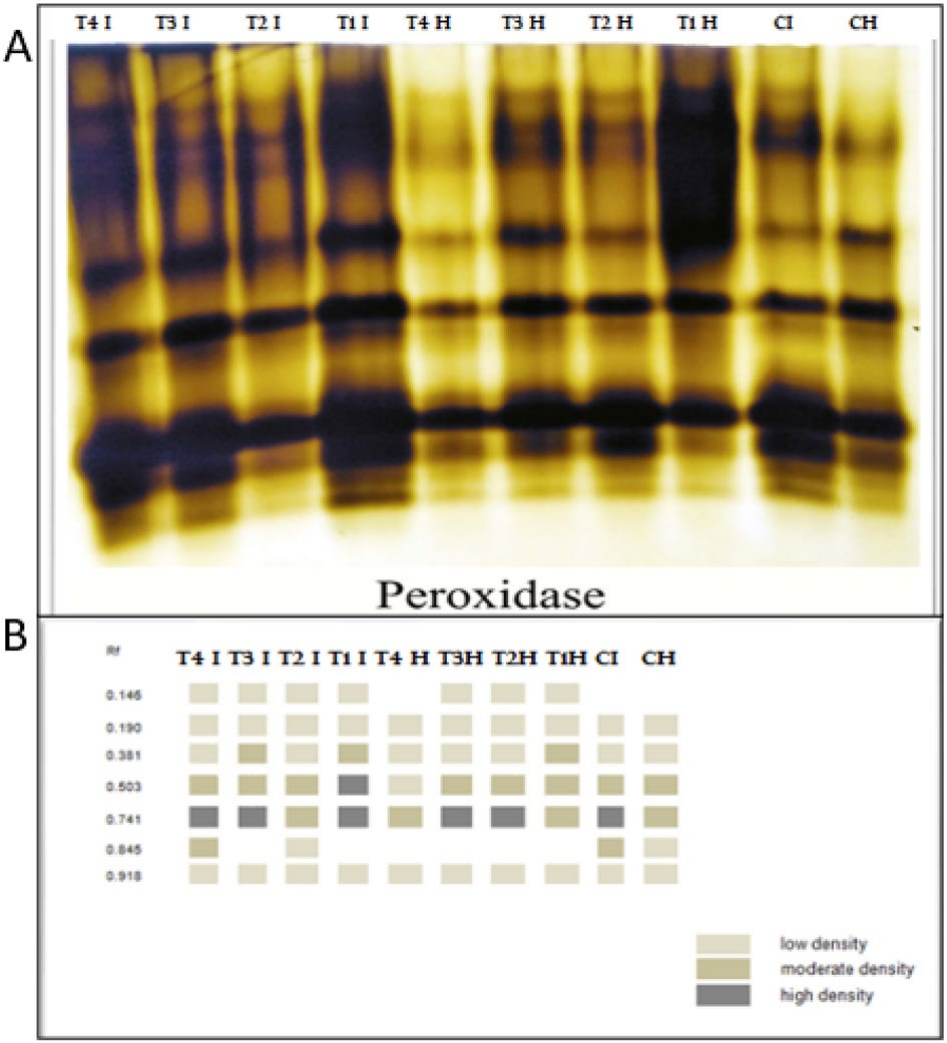
Consequences of *R. solani* infection, application of (*B. velezensis*, *B. megaterium,* and *H. huttiense)* and their impacts on tomato plants on (**A**) POD isozyme and (**B**) Ideogram analysis of POD isozyme of tomato plants.

Data offered in Fig. 13 and Table 5 showed seven POD isozymes at Rf 0.146, 0.190, 0.381, 0.503, 0.741, 0.845 and 0.918. *R. solani* infected plants displayed substantially overexpressed POD that documented 6 bands. Regarding infected plants, application of a combination of *B. velezensis*, *B. megaterium,* and *H. huttiense* revealed substantially overexpressed POD that detailed 7 bands followed by *B. megaterium* documented 7 bands, *H. huttiense* gave 6 bands and came next *B. velezensis* treatments documented the same 6 bands.

**Table 5:** Isomers of POD enzymes (+/−) and their Retention factor (Rf) in response to *R. solani*, application of (*B. velezensis*, *B. megaterium,* and *H. huttiense*) and their interactions on tomato plants.

RF	T4 I	T3 I	T2 I	T1 I	T4 H	T3 H	T2 H	T1 H	CI	CH
0.146	+	+	+	+	−	+	+	+	−	−
0.190	+	+	+	+	+	+	+	+	+	+
0.381	+	++	+	+	+	+	+	++	+	+
0.503	++	++	++	+++	+	++	++	++	++	++
0.741	+++	+++	++	+++	++	+++	+++	++	+++	++
0.845	++	−	+	−	−	−	−	−	++	+
0.918	+	+	+	+	+	+	+	+	+	+

CH = Healthy Control, CI = Infected control, T1H = Healthy + *B. velezensis*; T2H = Healthy + *B. megaterium*; T3H = Healthy + *H. huttiense*; T4H = Healthy + combination of (*B. velezensis*, *B. megaterium* and *H. huttiense*), T1I = infected + *B. velezensis*; T2I = Infected + *B. megaterium*; T3I = Infected + *H. huttiense*,; and T4I = Infected + combination of (*B. velezensis*, *B. megaterium* and *H. huttiense*).

### Polyphenol oxidase (PPO)

**Figure 14:**
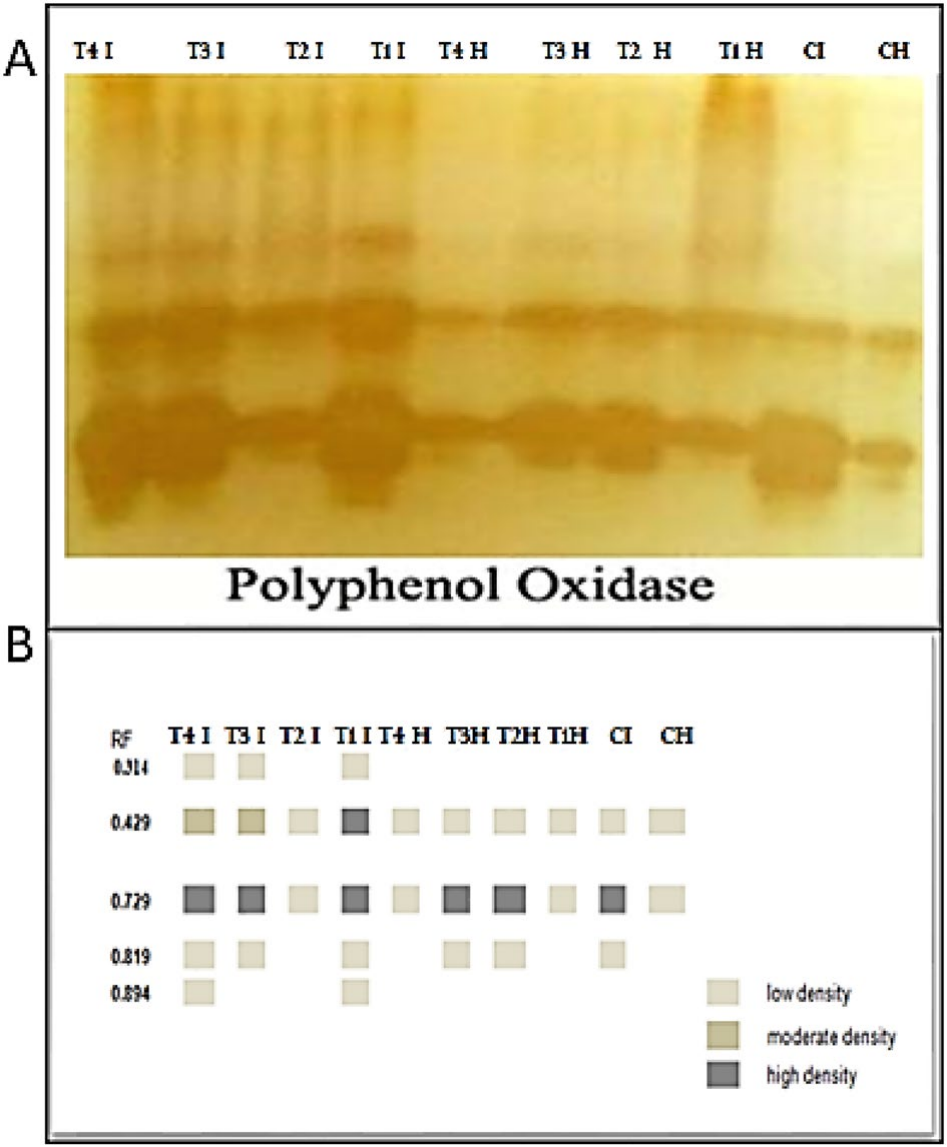
Consequences of *R. solani* infection, application of (*B. velezensis*, *B. megaterium,* and *H. huttiense)* and their impacts on tomato plants on (**A**) POD isozyme, and (**B**) Ideogram analysis of PPO isozyme of tomato plants.

Five PPO isozymes were visible on the native PAGE in Fig. 14 and Table 6. The infected plants revealed substantially overexpressed PPO 3 bands including 2 low bands at Rf 0.492 and 0.819, and a unique vastly dense band at Rf 0.732 related to healthy control. Additionally, under infection conditions, application *B. velezensis* revealed substantially overexpressed PPO that detailed two high dense and 3 low bands, followed by a combination of *B. velezensis*, *B. megaterium,* and *H. huttiense*, recorded the similar 5 bands at the identical Rf but the band at Rf 0.492 was moderate band, followed by *H. huttiense* recorded the same 5 bands two of them low at Rf 0.341and 0.819, while the other 1 moderate band was at Rf 0.49 and 1 high dense at Rf 0.732, and came next *B. megaterium* which recorded only two low bands at Rf 0.492 and 0.732.

**Table 6:** Isomers of PPO enzymes (+/−) and their Retention factor (Rf) in response to *R. solani*, application of *B. velezensis*, *B. megaterium,* and *H. huttiense*) and their interactions on tomato plants.

RF	T4 I	T3 I	T2 I	T1 I	T4 H	T3 H	T2 H	T1 H	CI	CH
0.341	+	+	−	+	−	−	−	−	−	−
0.492	++	++	+	+++	+	+	+	+	+	+
0.732	+++	+++	+	+++	+	+++	+++	+	+++	+
0.819	+	+	−	+	−	+	+	−	+	−
0.894	+	−	−	+	−	−	−	−	−	−

CH = Healthy Control, CI = Infected control, T1H = Healthy + *B. velezensis*; T2H = Healthy + *B. megaterium*; T3H = Healthy + *H. huttiense*,; T4H = Healthy + combination of (*B.velezensis*, *B. megaterium* and *H. huttiense*), T1I = infected + *B. velezensis*; T2I = Infected + *B. megaterium*; T3I = Infected + *H. huttiense*,; and T4I = Infected + combination of (*B.velezensis*, *B. megaterium* and *H. huttiense*).

## Discussion

Microorganisms are now used to control pathogens and pests to protect important plants^60^. The first and most important step in biological management is the identification and selection of active antagonistic microorganisms^61^. The goal of biological control is to sustain a balance in agrosystems in which the host suffers less damage in the presence of pathogens owing to the regulatory actions of non-pathogenic microorganisms that inhibit/antagonize plant pathogens. In the current work*,* endophytic bacteria *Bacillus velezensis* isolate L1, *Bacillus megaterium* isolate L2 and *Herpaspirillum huttiense* isolate S7 proved significant antifungal ability contrary to *R. solani* in Vitro. Our findings corresponded with those of Azevedo et al.^62^, who found that endophytic microorganisms have attracted attention for use in biological management and might be used to promote plant growth and prevent fungal infections. The isolated endophytic bacteria under study produce IAA, siderophores, HCN and solubilize phosphate in the soil. Indole acetic acid is a significant and powerful plant hormone, that controls cell growth and responds plants to light and gravity, as well as inducing plant growth and development^63,64^. Moreover, endophytic microorganisms produce bioactive compounds such as alkaloids, steroids, terpenoids, peptides, polyketides, flavonoids, quinols, and phenols. These compounds have a variety of agricultural, industrial, and therapeutic uses^30,65,66^. Endophytic bacteria have the ability to synthesize plant growth promotors, phosphate solubilization, mineral acquisition, and fixation of nitrogen^67^. Moreover, Shakirova^68^ indicated that IAA has been shown to play a significant fundamental function in cell division and root stimulation, which brings in improved plant development. HCN is a potent antifungal and has a significant role in the biocontrol of plant fungal pathogens^43,69^. Endophytes may have acted in various ways against plant pathogens, and this preliminary assessment suggests a potential ability to create metabolic substances that restrict pathogen growth or directly compete for nutrients and space, simulating conditions inside the plant that hosts them^70,71^. In addition, Schulz et al.^72^ proposed that endophytes are effective suppliers of secondary metabolites since they have an intimate connection to the host plants. Besides, Shukla et al.^73^ indicated that endophytes have been shown to produce a variety of bioactive compounds in a single plant or microbe, making them a promising source of drugs for the treatment of various diseases, as well as having conceivable uses in agriculture. The endophytes' production of bioactive compounds has been linked to the advancement of the host microbes, which might have adopted genetic codes from higher plants, thus allowing them to more easily adapt to their host plant and fulfill certain roles, such as protection from numerous sorts of pathogens^74,75^.

Similar to our findings, Devi et al*.*^76^ reported the usage of *B. velezensis* as a biocontrol agent has a wide antifungal spectrum and affords a sustainable alternative for hazardous chemicals. Besides, our results agreed with Hashem et al.^48^ who recorded the antifungal potential of *B. megaterium* against phytopathogenic *R. solani*. It's interesting to note that, except for a few isolates rarely reported in humans as a pathogen causing bacteremia in leukemic persons^77,78^, the majority of the *Herbaspirillum* genus were mostly isolated from soil, plants, or water. The isolation of the novel endophytic bacterium *Herbaspirillum camelliae sp.* from *Camellia sinensis L.* was also described by Liu et al.^79^. The study of Andreozzi et al.^80^ showed the beneficial endophyte *Herpaspirillum huttiense* RCA24 as a promising strain to improve rice plants in the greenhouse. Endophytic actinomycetes and bacteria are important contributors to the synthesis of bioactive substances. Moreover, Liarzi et al.^81^ reported that endophytic bacteria endure most of their lives inside plant tissues without causing any evident impairment to the host plant and improve plant tolerance to various abiotic and biotic stress, as well as plant durability against pests and insects.

The combination of endophytic bacteria under study increased the tomato's protection against *R. solani* disease by 79.4%, according to the results. *B.* velezensis has been shown to exert opposing effects on plant diseases by producing a variety of antimicrobial chemicals; siderophore bacillibactin^82–84^.

Besides, *R. solani* infectivity causes significant decreases in the contents of soluble carbohydrates and soluble proteins in our study. *R. solani* caused reduced photosynthetic rate and excessive respiration rate in infected plants resulting in lower concentrations of both soluble carbohydrates and protein^85–87^.

The results also showed that the application of (*B. velezensis, B. megaterium*, and *H. huttiense*) individually or in combinations had a substantial impact on the level of osmolytes (total soluble sugar and soluble protein), which may operate as a marker of resistance, in both healthy and diseased plants. The noticed increase in tomato plant soluble sugar and protein could be attributed to the ability of these endophytic bacteria to fix nitrogen, produce plant growth regulators; and solubilize phosphate, which enhances nutrient uptake from the root rhizosphere^88,89^.

The phenol content increased by 52.77%, due to *Rhizoctonia solani* infection in comparison with healthy control. These results are supported by many previous findings^26,90^. The role of phenolic compounds is fundamental in preventing the spread of root rot diseases by either making numerous metabolic products including those involved in host defense mechanisms, or by reducing the pathogen's toxicity and increasing host defense pathways^91^. Besides, phenolic chemicals were able to strengthen cellular membranes by restricting membrane flexibility, which lowers the ability of free radicals to cross membranes and causes membrane peroxidation^92,93^. Our results were supported by many previous findings^48,94^.

Herein, *R. solani* infection accumulated MDA by 57.6% and H_2_O_2_ by 140%, compared to untreated healthy plants. These results were supported by previous findings^87,95^. By boosting antioxidant molecules that eliminate ROS and protect cell membranes against oxidative stress, the application of endophytic bacteria reduced the generation of MDA and H_2_O_2_^96^. One of the most important signs of stress resistance is avoidance and reduction of oxidative stress and capture of free radicals^97^.

Our findings confirmed that plants subjected to *R. solani* infection had considerably higher activity levels of antioxidant enzymes. The plant displayed various defense mechanisms against infection, boosting the action of antioxidant enzymes to maintain ROS levels in plant cells low. POD and other antioxidant enzymes assist in converting H_2_O_2_ to H_2_O^98^. Increasing the activity of antioxidant enzymes provides a key function in plant physiological immunity and defending cells from oxidation as a result of infection^99^. As part of the primary regulating mechanisms of metabolism within cells, several enzyme isoforms are essential for plant cellular defense versus biological stress^100,101^. The generation of these isozymes is thought to be essential for the cell's defense against oxidative damage^102,103^. In the leaf-soluble protein extracts of the tomato plant, active staining of antioxidants disclosed five PPO isozymes, seven SOD isozymes, and seven POD isozymes. The antioxidant enzyme levels in *R. solani*-infected plants handled with endophytic bacteria *B. velezensis, B. megaterium,* and *H. huttiense* were greater than in the control one since numerous unique bands were produced because of infection. The stressed tomato plants treated with *B. velezensis, B. megaterium, and H. huttiense* either in combination or alone displayed the highest bands of POD isozyme allied to another treatment. The outcomes demonstrate how treatments protect tomato plants from root rot disease in an ameliorative manner which is agreed with Hao et al.^104^. These findings are consistent with the findings of Rajendran et al.^105^, which reported increased antioxidant isozyme transcript abundances in plants. In comparison to untreated and infected control plants, the level of all SOD isozymes intensified when endophytic bacteria (*B. velezensis, B. megaterium,* and *H. huttiense*) were applied. The recent results were in the same link as prior investigations that claimed a range of proteins, including CAT and POD, may act as scavengers for these ROS^106,107^.

## Conclusion

The present investigation used a promising approach that was focused on applying endophytic bacteria to promote systemic resistance in tomato plants against *Rhizoctonia* root-rot disease. Three endophytic bacteria; *Bacillus velezensis* OQ073573, *Bacillus megaterium* OQ073583*,* and *Herpaspirillum huttiense* OQ073584 isolated from strawberry plants boost the systemic resistance in tomato plants and reduce the severity of the *Rhizoctonia* root-rot disease. These endophytic bacteria demonstrated potent antifungal activity against *R. solani* in vitro along with in vivo. Pre-treated tomato plants with endophytic bacteria had significantly higher levels of total soluble proteins, total carbohydrates, and phenols. Interestingly, the harmful impact of *Rhizoctonia* root-rot disease on tomato plants was pointedly decreased and it can be clear from diminished MDA and H_2_O_2_ levels. In considering this, endophytic bacteria are promising isolates for application in agriculture, as an effective biological manager against *Rhizoctonia* root-rot, and for the induction of healthy tomato plants.

## Data Availability

The datasets generated and/or analyzed during the current study are available in the Gene Bank database repository, under Accession No. OQ073573 for *Bacillus velezensis*, GenBank accession OQ073583 for *Bacillus megaterium and* GenBank Accession No. OQ073584 for *Herpaspirillum huttiense.*
